# The Effect of Medicines upon the Teeth

**Published:** 1876-05

**Authors:** J. F. P. Hodson


### ARTICLE VII.

### *The Effect of Medicines Upon the Teeth.*

BY J. F. P. HODSON, D. D. S.

It is an assertion very frequently made by patients to their dentist, in excuse for a largely decayed or much filled set of teeth, or perhaps to account for their entire absence, that at such or such a time they had a severe illness, and that their physician had ruined their teeth with powerful medicines. This is a very popular and deep-seated impression, and prevailing as it does among the most intelligent people, as well as those of other classes, it cannot be properly denominated a vulgar superstition or an ignorant prejudice, for the basal principle upon which they have built their pet theory is, alas, *very* true,—the teeth *have* decayed during the illness, and in some instances even more than during all the previous life taken together. I am persuaded, however, that as to the cause, the allegation is in the great majority of cases, untrue, and that the exhibition of acid remedies had comparatively little to do with the matter, and that this little may be reduced to a mere shadow by proper care. I hope to hereinafter show, as well as to offer some suggestions that may prove of service to the physician in warding off from the teeth of his helpless and practically unconscious patient, some of the terribly destructive influences which obtain during this period.

During the latter months of the period of gestation, and in fevers very especially, or the progress of any illness involving a feverish condition the fluids of the mouth are constantly as intensely acid, as respects the teeth, as is any medicine administered by the physician; and, moreover, from the high temperature of the buccal cavity at such times, the power of these acids for evil is greatly augmented Further, a direct consequence of these conditions is the especially rapidly fermentation and decomposition of all food lodged between and around the teeth, and the consequent elimination of other deleterious acids,—all forming a mighty power, under which it is not surprising that tooth-structure should melt away like dew before the sun. It is indeed a very surprising thing that any tooth should live to tell the tale.

Still further, during such a period and condition of things —when of all times in one's life the mouth needs the best and most faithful care, it, in reality, because of the helplessness of the patient, receives *absolutely none*, and the teeth, poor victims, are left entirely at the mercy of this grand coalition of their enemies, who are all eager to advance their work of destruction, aye, complete it before they shall have again been brought to bay by the conditions of returning health and the tooth brush of the patient. And all this, forsooth, the physician's "powerful medicines" accomplish.

The physician is, however, the strong right arm upon which the patient should lean during illness, at least as to all things directly pertaining to it. To the physician, therefore, I appeal in behalf of all our mutual patients, that they may, by a few timely suggestions, looking to the neutralization of the acid condition then prevailing in the oral cavity, very essentially reduce the dreadful ravages of this period upon the teeth—such, for instance, as directing the patient to rinse the mouth frequently with *liquor calcis*, diluted according to the sensitiveness of the mucous membrane, and flavored with a few drops of wintergreen or peppermint to make it agreeable; or to rub *creta prep.* freely about and between the teeth, and allow it to remain during the night; or, if the patient be able to use the tooth-brush, to add a small quantity of *soda btcarb.* to the usual tooth-powder (for this purpose only, and not for general use;) or, in short, the employment of any of the manifold methods of neutralizing the acid condition which may be most accessible at the time.

It may be objected to the foregoing that the mouth, during the progress of a fever, contains but little fluid of any kind; true, but teis very circumstance makes the little which is there—the mucous principally—especially concentrated, and correspondingly baneful in its effects upon the teeth; whereas in a normal condition of the system, the the acidity of the mucous is entirely neutralized by the large influx of the alkaline saliva.

The tube through which acid remedies are usually directed to be taken does not prevent them from regurgitating upon the teeth in the act of swallowing, so that it practically accomplishes very little of the object sought to be attained by its employment; but, if in addition the mouth be immediately and thoroughly rinsed with the solution of lime above referred to, or a solution *sup cnrb. soda*, the evil effects of any acid remedy upon the teeth will be entirely obviated.

In the exhibition of strong alkalies, on the other hand, which lay hold of the gelatinous constituents of the teeth, the fluids of the mouth during illness are, as has been stated, usually so pronouncedly acid as to entirely neutralize this condition ; but if otherwise, a slight rinsing with a very dilute simple acid, like tartaric or citric, will accomplish the result desired, though the effect of these remedies upon the teeth is scarcely worthy of consideration, so entirely will the matter of their neutralization take care of itself. And even at other times than during actual illness is this the case with many of our people, who with their irregularities, hot foods and drinks, the precipitate haste of their eating, and the half-masticated masses with which their stomachs are constantly insulted, are, even in health (?) much addicted to gastric disturbances, acid eructations, depraved and vitiated secretions, and a generally dyspeptic diathesis.—than which condition, by the way, none could be more inimical to the integrity of the dental structures—*Med. Record.*

---

